# Mental health systems in six Caribbean small island developing states: a comparative situational analysis

**DOI:** 10.1186/s13033-022-00552-9

**Published:** 2022-08-12

**Authors:** Ian F. Walker, Laura Asher, Anees Pari, Jennifer Attride-Stirling, Ayoola O. Oyinloye, Chantelle Simmons, Irad Potter, Virginia Rubaine, June M. Samuel, Aisha Andrewin, Janett Flynn, Arline L. McGill, Sharra Greenaway-Duberry, Alicia B. Malcom, Gemma Mann, Ahmed Razavi, Roger C. Gibson

**Affiliations:** 1 Public Health England, Wellington House, 133-155 Waterloo Road, London, SE1 8UG UK; 2 University of Nottingham, School of Medicine, Academic Unit of Population and Lifespan Sciences, Nottingham, UK; 3 Ministry of Youth, Culture and Sport, Government of Bermuda, Hamilton, Bermuda; 4 Department of Health, Government of Bermuda, Hamilton, Bermuda; 5 Bermuda Hospitals Board, Devonshire, Bermuda; 6 Ministry of Health and Social Development, Government of the Virgin Islands, Tortola, British Virgin Islands; 7 Health Services Authority, Government of the Virgin Islands, Tortola, British Virgin Islands; 8 Ministry of Health and Social Development, Government of Anguilla, The Valley, Anguilla; 9 Ministry of Health, Environment, Culture and Housing, Cayman Islands Government, Grand Cayman, Cayman Islands; 10 Health Services Authority, George Town Hospital, Cayman Islands Government, Grand Cayman, Cayman Islands; 11 Ministry of Health & Social Services, Government of Montserrat, Little Bay, Montserrat; 12 Ministry of Health, Agriculture, Sports and Human Services, Government of the Turks and Caicos Islands, Grand Turk, Turks and Caicos Islands; 13 Department of Community Health & Psychiatry, The University of the West Indies, Mona Campus, Kingston 7, Jamaica

**Keywords:** Small island developing states, Caribbean region, Mental health systems, Mental health services

## Abstract

**Background:**

Small island developing states (SIDS) have particular mental health system needs due to their remoteness and narrow resource base. We conducted situational analyses to support mental health system strengthening in six SIDS: Anguilla, Bermuda, British Virgin Islands, Cayman Islands, Montserrat and Turks and Caicos Islands.

**Methods:**

The situational analyses covered five domains: 1. Socio-economic context and burden of mental disorders, 2. Leadership and governance for mental health 3. Mental health and social care services 4. Strategies for promotion and prevention in mental health and 5. Information systems, evidence and research for mental health. First, a desk-based exercise was conducted, in which data was drawn from the public domain. Second, a field visit was conducted at each site, comprising visits to facilities and consultation meetings with key stakeholders.

**Results:**

Our key findings were 1. Despite most of these SIDS being high-income economies, social inequalities within states exist. There was no population-level data on mental health burden. 2. All SIDS have a mental health policy or plan, but implementation is typically limited due to lack of funds or staff shortages. There was minimal evidence of service user involvement in policy or service development. 3. All SIDS have a specialist, multi-disciplinary mental health workforce, however Montserrat and Anguilla rely on visiting psychiatrists. Child and adolescent and dedicated crisis intervention services were found in only two and one SIDS respectively. A recovery-oriented ethos was not identified in any SIDS. 4. Mental illness stigma was prevalent in all SIDS. Promotion and prevention were objectives of mental health strategies for all SIDS, however activities tended to be sporadic. No mental health non-Governmental organisations were identified in three SIDS. 5. Health information systems are generally underdeveloped, with paper-based systems in three SIDS. There has been no rigorous local mental health research.

**Conclusion:**

Cross-cutting recommendations include: to develop mental health action plans that include clear implementation indicators; to facilitate community surveys to ascertain the prevalence of mental disorders; to explore task-sharing approaches to increase access to primary mental health care; and to develop programmes of mental health promotion and prevention.

**Supplementary Information:**

The online version contains supplementary material available at 10.1186/s13033-022-00552-9.

## Background

Mental and addictive disorders represent an important and growing contributor to disease burden globally. In 2016 they caused 7% of all global burden of disease and 19% of all years lived with disability [1]. Suicide was the second leading cause of death in young people aged 15–29 years for both sexes in 2016 [2]. Public mental health is also highly influenced by global events, such as the Covid-19 pandemic and increasing international migration [3, 4]. Yet despite the increasing burden of mental illness, mental health systems are a low priority in most countries globally.

Small island developing states (SIDS) are recognised to have particular vulnerabilities and development needs due to their small size, remoteness, and susceptibility to climate change impacts [5]. The small population size and narrow resource base in terms of human resources and facilities mean that SIDS tend to be deprived of the advantages of the economies of scale [5, 6]. Reduced internet connectivity, shortage of medicines and high production costs may also impede the development and running of high-quality health systems [7]. Together these factors are understood to create substantial barriers to the provision of universal health coverage in many SIDS [6]. These settings may also have particular mental health needs. The loss of life and threats to livelihoods associated with frequent hurricanes in the Caribbean region have significant mental health sequelae [8, 9], whilst rising sea levels related to climate change are a source of fear and worry amongst residents of coastal regions in some SIDS [10, 11]. The lack of anonymity and privacy experienced in small populations could have important mental health impacts. An analysis of Bermudian print media found many articles relating to mental illness included photographs and personally identifiable information. It was hypothesised that fear of public exposure in the national press could prevent some service users from accessing care and therefore impede recovery [12]. Furthermore, the increased potential for social isolation on remote islands could conceivably heighten established associations between loneliness and mental health outcomes [13, 14]. Conversely, membership of the small, tight-knit communities often found on SIDS may protect against social isolation and improve wellbeing.

Mental health systems, comprising the organisations, institutions and resources whose purpose is to improve mental health, should be responsive to local needs. Whilst many SIDS have high-income economies, limited access to specialist mental health providers and facilities means that some of the mental health systems challenges they face are more familiar to low- and middle-income countries [15]. Small island settings may encounter particular difficulties in meeting international standards relating to recovery and care; for example in relation to compulsory treatment and detention, and providing the long term intersectoral rehabilitation services needed to meet the complex health, social and economic needs of persons with chronic mental disorders [16, 17]. A lack of available and accessible mental health services may increase inequalities in mental health outcomes within SIDS.

The World Health Organisation’s Mental Health Action Plan 2013–2030 sets out global priorities for addressing the mental health burden. Core objectives relate to improving leadership and governance, community-based mental health and social care services, promotion and prevention in mental health and information systems and evidence [18]. Mental health promotion and prevention efforts are increasingly emphasised as the most sustainable and impactful means of improving public mental health [19]. The action plan recommends pursuing a cross-cutting approach to mental health system strengthening in order to address these objectives and ultimately improve mental health outcomes. Yet research and guidance relating to mental health systems strengthening in SIDS settings is lacking.

Initiatives such as the SIDS Accelerated Modalities of Action (SAMOA) Pathway, which was endorsed by the UN General Assembly in 2014, have sought to address development needs and priorities of SIDS [5], but tend not to specifically tackle mental health. Whilst the SAMOA pathway recognises mental wellbeing, along with physical and social wellbeing, as essential for the achievement of sustainable development, recommended actions focus on communicable diseases such as malaria; non-communicable diseases; maternal and child mortality; and broader public health endeavours such as healthy diet. Although some mental health issues may be subsumed under non-communicable diseases, a more comprehensive approach is needed. Many Caribbean states have, in fact, included mental and substance use disorders in their broader efforts to tackle non-communicable diseases. However, the disruption to healthcare services and prevention efforts caused by the COVID-19 pandemic is likely to have substantially hindered these endeavours, halting previous progress [20].

Situational analysis is ‘an assessment of the current health situation and is fundamental to designing and updating national policies, strategies and plans’ [21]. Components of a situational analysis may include human, financial and institutional resources, health services, existing health policies and plans, monitoring and evaluation, intersectoral collaboration, service user needs and characteristics and the social determinants of health [22]. A situational analysis aids the early consideration of these factors, and how they interact, and therefore promotes the design of realistic, contextually appropriate policies and programmes that are likely to be feasible, acceptable and effective in the local setting. This paper presents findings of situational analyses conducted to support mental health system strengthening in six SIDS: Anguilla, Bermuda, British Virgin Islands (BVI), Cayman Islands, Montserrat and Turks and Caicos Islands (TCI). By comparing contextual factors and resources we aimed to facilitate shared learning and policy development across the six settings.

## Methods

### Study context

Anguilla, BVI, Cayman Islands, Montserrat and TCI are located in the Caribbean (Table 1). Bermuda lies in the North Atlantic Ocean approximately 1500 km north of the Caribbean. Anguilla and Montserrat are single islands whilst the Cayman Islands comprises three islands. Bermuda, BVI, and TCI are archipelagos made up of large numbers of small islands. Bermuda’s seven main islands are linked by bridges. In other states air travel is a common form of transportation between islands [23].

**Table 1:** Setting, socio-economic context and burden of mental disorders

Indicator	Anguilla	Bermuda	BVI	Cayman Islands	Montserrat	TCI
Location & geography	Single island 91 km^2^ Eastern Caribbean	Archipelago of eight main islands & > 100 smaller islands (8 inhabited) 53 km^2^ Atlantic Ocean. 1500 km north of the Caribbean	Archipelago of five main islands & 50 smaller islands (16 inhabited) 151 km^2^ North Eastern Caribbean	Three islands (all inhabited) 264 km^2^ Western Caribbean	Single island 102 km^2^ Eastern Caribbean	Archipelago of 40 islands (8 inhabited) 616 km^2^ Northern Caribbean
Total population	15, 000 (2020 estimate) [54]	63, 908 (2019 estimate) [55]	30, 000 (2020 estimate) [56]	69, 914 (2019 estimate) [57]	4, 639 (2018 Labour Force Survey) [58]	44, 543 (2020 estimate) [59]
GDP per capita	US$25, 529 (2019) [60]	US$117,768 (2019) [61]	US$43,189 (2019) [62]	US$92, 692 (2019) [63]	US$13, 483 (2019) [64]	US$31, 353 (2019) [65]
Life expectancy at birth	81.6 years (2013) [66]	82.8 years (2020) [67]	79.9 years (2015) [68]	81.2 years (2013) [69]	74.4 (2013) [70]	79 years (2019) [59]
Ethnicity	African/black: 85.3% White: 3.2% Hispanic: 4.9% Mixed 3.8% Asian: 1% Other 1.9% (2011) [71]	Black: 58.9% White & other: 40.9% (2016) [55, 72]	African/black: 76.3% White:5.4% Latino: 5.5% Mixed: 5.3%, Other:7.5% (2010) [73]	Black: 20% White: 20% Mixed: 40% Other: 20% 2010 [52]	African/black: 86.2% White: 2.7% Hispanic: 3% Mixed: 4.8% Asian: 1.6% Other: 1.4% (2018) [58]	Black: 83.9% Hispanic: 5.3% White: 4.7% Mixed 1.9%, Asian: 0.7% Other: 1.2% (2012) [74]
Non-belonger or expatriate populations	Data not found	Bermudian: 79% Non-Bermudian: 21% (2016) [55] Top ranked country of origin for non- Bermudian: UK, US, Canada, Jamaica, Philippines [55]	BVI born: 39.1% Overseas born: 60.9% (2010) [73] Top ranked country of origin for non-BVI born: Guyana, St. Vincent and Grenadines, Jamaica, US, Dominican Republic [73]	Caymanian: 53.4% Non-caymanian: 46.4% (2019) [57] No country of origin data found	Monserratian: 63% Non-Montserratian: 37% (2018) [58] Top ranked country of origin for non- Montserrat born: Guyana, Jamaica, UK and Dominica	Belonger: 40% Non-belonger: 60% [75] Top country of origin for non- TCI born: Haiti, Dominican Republic, Jamaica, Guyana, Philippines
Unemployment	13% (2011) [71]	3.8% (2019) [76]	2.9% (2015) [77]	3.5% (2019) [57]	6.5% (2018) [58]	7% (2019) [59]
Burden of mental health problems in children and adolescents	Global School Health Survey 2016 (age 13–17; n = 813): Suicidal ideation 30.5% (95% CI 28.2 to 32.9) Suicide attempt 19.4% (95% CI 17.5 to 21.5)[30]	Health Survey of Bermuda 2006 (age 4–10; n = 196): Not well behaved 3.1% Many worries 5.7% Unhappy 2.6% Poor attention span 4.7% [78]	Global School Health Survey 2009 (age 13–15) Suicidal ideation 15.7% Suicide attempt 12.5% [31]	Global School Health Survey 2007 (age 13–15): Suicidal ideation 19.0% [29] Student Drug Use Survey 2016 (years 9–12; n = 1800) 68% self-rated mental health as ‘good’[32]	Global School Health Survey 2008 (age 13–15; n = 212): Suicidal ideation 16.4% [28]	No data found
Burden of mental health problems in adults	No data found	Health Survey of Bermuda 2016 (n = 802): Self-reported lifetime depressive disorder 14.0% [33]	No data found	No data found	No data found	No data found

The six SIDS are all self-governing UK overseas territories (UKOTs). Since 2018 Public Health England (PHE) has delivered a programme of public health technical assistance with the UKOTs, funded by the Foreign and Commonwealth Office. The work presented here was conducted at the request of each of the respective Ministries of Health (MoH), as part of a PHE work package focused on mental health systems strengthening.

### Scope of situational analysis

The situational analysis covered five domains, which map onto the WHO Mental Health Action Plan 2013–2030 objectives.

1. Socio-economic context and burden of mental disorders
2. Leadership and governance for mental health (mental health policy, legislation and financing; and service user involvement)
3. Mental health and social care services (mental health human resources and facilities, including primary care and specialist services; treatment coverage)
4. Strategies for promotion and prevention in mental health (mental health literacy & attitudes; suicide prevention; promotion and prevention activities including schools; intersectoral working)
5. Information systems, evidence and research for mental health.

### Data collection

Data was collected from BVI in October–November 2018; from Anguilla in June–July 2019; from Bermuda in August–September 2019; from TCI in October–November 2019; from Montserrat in November–December 2019 and the Cayman Islands in February–March 2020. To guide data collection we developed a bespoke situational analysis tool covering the five domains (see Additional file 1), drawing on international best practice from the Programme for Improving Mental healthcare (PRIME) situational analysis tool [24] and the WHO Assessment Instrument for Mental Health Systems [25].

For each SIDS, data collection was completed in two stages. First, a desk-based exercise was conducted, in which data was drawn from the public domain and entered into the situational analysis tool. Sources included health surveillance data, census reports, policy documents, governmental and non-governmental reports, Pan American Health Organisation (PAHO) country profiles and the WHO Mental Health Atlas. Additional material for the desk-based review was also solicited from the MoHs and other stakeholders, e.g. internal reports and draft documents. We searched Medline using the terms “Psychiatry”, “Mental health”, “Health services”, “Public health” or related terms and “Small Island developing state” or each included SIDS. Second, a 5-day field visit was conducted at each site by a small team (2–4 persons) of UK specialists in public mental health and, for three SIDS, an external consultant psychiatrist advisor from the Caribbean region. They comprised of visits to facilities and institutions, and consultation meetings with key stakeholders. Stakeholders were identified in consultation with the Chief Medical Officer for each SIDS, and included directors of key services (healthcare, social services, prison, police, youth affairs and Non-Governmental Organisations (NGOs) e.g. Red Cross), mental health service users, health planners, health promotion leads, general practitioners, psychiatrists, psychiatric nurses, psychologists, social workers, prison officers, public health nurses, community mental health officers, school nurses, counsellors, clergy, teachers and care workers. We used the situational analysis tool headings to guide consultation meetings, focusing on validating information gathered during the desk-based exercise and gathering new data where gaps existed. We tried to establish the trustworthiness of data from grey literature by triangulating data sources where possible. All draft findings were reviewed for accuracy by the CMO, and other representatives in the Ministry of Health, for each SIDS before finalising.

## Results

### Socio-economic context and burden of mental disorders

The total population of these SIDS ranges between 5000 (Montserrat) to 69,900 (Cayman Islands) (Table 1). The main economic activities are tourism and offshore financial services. There are substantial differences in GDP per capita between them, ranging from $13,483 in Montserrat to $117,768 in Bermuda. Life expectancy at birth shows a similar pattern, with estimates spanning 74.4 years in Montserrat to 83.5 years in Bermuda. Unemployment rates are generally low, with the lowest rates in BVI (2.9%) and highest rates in Anguilla (13%) and Montserrat (7%). Most are ethnically diverse. Anguilla, Montserrat and TCI have the greatest proportion of black population (85–87%), and Cayman Islands has one of the lowest (20%). People of Asian, Hispanic, mixed and White ethnic origin make up smaller proportions of the population.

Belonger status is a legal classification unique to the UKOTs that indicates an individual has close ties to a specific territory, normally by birth or ancestry. The requirements for belonger status, and the rights that it confers, vary from territory to territory.

Non-belongers form the majority of the population in TCI (60%) and a substantial minority in other UKOTs (e.g. 46.4% in Cayman Islands, 33.8% in BVI). Country of origin of non-belongers varies by UKOT but includes other Caribbean countries (including Haiti, Jamaica and the Dominican Republic), UK, USA, Guyana and the Philippines.

Despite most of these SIDS being high-income economies, social inequalities within states do exist. For example, in 2012 a Country Poverty Assessment found that 40% of residents in the Caicos islands group live in poverty (defined as income less than $18.2/day), compared to 17% on Providenciales, another island of TCI. There are also indications that disadvantage is concentrated in certain ethnic groups and nationalities of origin. In TCI, Haitian nationals had the highest poverty rates, at 35%, compared to the national average of 22% [26]. In Bermuda, 52% of the white population had a university degree compared to 35% of persons of mixed and other races and 26% of the black population. Black persons also had substantially lower incomes than white persons at all education levels, and higher rates of unemployment [27].

In 2017 Hurricanes Irma and Maria caused substantial damage to Anguilla, BVI and TCI. Bermuda, Montserrat and the Cayman Islands are also occasionally affected by hurricanes. Where WHO Global School-based Student Health Surveys had been conducted, (Anguilla, BVI, Cayman and Montserrat) there were reportedly high levels of suicidal ideation among adolescents (range 12.5% to 30.5%) [28–31]. However the validity of this data was uncertain and surveys were conducted up to 14 years ago, so may not reflect current burden. The 2016 Cayman Islands Student Drug Use Survey found that 68% of students in Year 9–12 rated their mental health as ‘good’ [32]. In a 2011 population health survey of adults in Bermuda self-reported lifetime depressive disorder was 14.0% [33]. No other data on prevalence of mental disorders was identified for any included SIDS.

### Leadership and governance for mental health

Five of the six SIDS (Anguilla, BVI, Cayman Islands, Montserrat and TCI) have a dedicated mental health policy or strategy, all of which were created in the last seven years and assessed to align with international human rights standards (Table 2). Despite this, only Montserrat has an active mental health action plan. Anguilla, BVI and TCI have action plans under development or in draft form. Whilst Bermuda does not have an overarching mental health policy, in 2010 the Bermuda Hospital Board prepared a mental health plan focusing on mental health service improvements. At the time of the situational analysis, an updated plan was also being pursued. Mental health policies and plans, where they exist, were found to have been only partially implemented or not at all. For example, objectives related to mental health promotion and prevention had not been met in BVI or Montserrat, whilst some planned service developments, such as the relocation of long-stay inpatient and outpatient services from the mental hospital to community settings in Bermuda, had stalled. Implementation issues were largely due to lack of funds and political prioritisation, suitable facilities and relevant staff. No SIDS was found to have clear mechanisms for monitoring implementation. There were some examples of integration of mental health into broader policy and planning, such as the mental health components of TCI’s disaster management plan and Bermuda’s health promotion strategy. However, there were also notable absences of mental health, for instance BVI’s non-communicable disease strategy. None of the SIDS had a distinct mental health authority responsible for mental health delivery. All but Montserrat had a health service authority separate to the Ministry of Health, within which the public mental healthcare services were provided.

**Table 2:** Leadership and governance for mental health

Indicator	Anguilla	Bermuda	BVI	Cayman Islands	Montserrat	TCI
Mental health policy/ strategy	Yes 2013	No	Yes 2013	Yes 2017	Yes 2015–18	Yes 2015
Mental health action plan	Yes 2013	Yes 2010	Yes (draft) 2016	No	Yes 2015–18	No
Policy/ plan in line with international human rights	Yes	Yes	Yes	Yes	Yes	Yes
Policy/ plan implementation	No	Limited	Limited	Limited	No	Limited
Inclusion of mental health in broader health policies	Partial- national health framework 2013	Yes- Well Bermuda (health promotion) strategy	No	No	No	Yes- Disaster Management Plan and Health Sector Plan
Involvement of people with lived experience	No	No	No	Partial	No	No
Mental health legislation	Yes 2006	Yes MH Amendment Act 2019. Mental Health Act Code of Practice 2021	Yes 2014	Yes 2013	Yes 2013	Yes 2016
Involuntary treatment included in mental health legislation	In hospital—yes In community—no	In hospital—yes In community—yes	In hospital—yes In community—yes	In hospital—yes In community—yes	In hospital—yes In community—no	In hospital—yes In community—no
Health service financing	Government finance, voluntary health insurance, out-of-pocket expenditure	Government finance, compulsory/ voluntary health insurance, out-of-pocket expenditure and donor financial assistance	Government spending, compulsory/voluntary health insurance, out-of-pocket expenditure and donor financial assistance	Government finance, compulsory/voluntary health insurance, out-of-pocket expenditure	Government finance (free at point of care)	National health insurance (free at point of care)

In general, there was very limited evidence of service user involvement in policy or service development. An exception to this is in the Cayman Islands, where people with lived experience are represented in the Mental Health Commission, a body established in statute, which makes recommendations to government and service providers on ways to improve the local mental health system. Intersectoral collaboration with a focus on mental health, for example with education, housing and social welfare, was generally weak in all SIDS at both government department and service provision levels.

With the exception of Bermuda, all UKOTs have a Mental Health Act passed in the last 15 years. Bermuda’s Mental Health Act 1968 underwent an amendment in 2019 and in line with this, second opinion approved doctor reviews were introduced and a new Mental Health Act Code of Practice was published in 2021 (after completion of the situational analysis). Legislation in all the SIDS contained provisions for involuntary detention and treatment in hospital and for Bermuda, Cayman Islands and BVI the legislation also included involuntary treatment in the community. However, it was noted that some of these SIDS contained provision for prisons to be ‘places of safety’ for treatment of those with mental disorders, which was a concern.

The proportion of the health budget spent on mental health was generally difficult to obtain. In most UKOTs the majority of mental health expenditure related to services delivered in tertiary settings catering for people with severe mental disorders. In most SIDS mental health spending was financed by a combination of government finance, compulsory and voluntary health insurance and out-of-pocket expenditure. Montserrat benefits from UK Government donor financial assistance for some of its healthcare costs. The relative importance of different funding sources varied; for instance, a high proportion of overall healthcare spending in Anguilla is out-of-pocket expenditure (estimated at 60–70%). Several stakeholders, including health and social care professionals and service users, highlighted out of pocket payments as a source of emotional and financial stress and a barrier to accessing care. Even where mental health financing is dominated by national insurance programmes that provide universal coverage for basic health care to all residents linked to employment, mental healthcare is often not covered to the same extent as physical healthcare. Means tested support is also available for those not covered through these schemes in most SIDS; however, immigrants are not included (apart from Bermuda), so have to pay out of pocket for any healthcare expenses incurred. All SIDS have the facility, on application, for the government to cover transport to a larger island for specialist treatment for those on low-income. In TCI, individuals with a mental disorder can apply to the Department of Social Development for assistance with medical fees.

### Mental health services

All these SIDS have a specialist, multi-disciplinary workforce in mental healthcare, however expertise and staffing varied between SIDS (Table 3). Whilst all have psychiatrist input, psychiatric nurses and psychologists, only Bermuda, BVI, Cayman Islands and TCI have mental health social workers, mental health officers or psychiatric aides. Montserrat and Anguilla rely on visiting psychiatrists who are not permanently on island; and normal services have been disrupted due to the COVID-19 pandemic. By contrast the Cayman Islands and Bermuda have three and five public sector consultant psychiatrists, respectively. All these SIDS have dedicated inpatient acute facilities, apart from Montserrat which has one side room on a ward that in practice is rarely used for mental health service users. Those with dedicated units have an acute bed capacity of between eight and 23 beds; most are single rooms but Anguilla uses small dormitories. All reported some long-stay inpatients using acute beds, occasionally for several years, due to inadequate accommodation in the community. In most SIDS only a small number of service users detained under mental health laws reportedly appealed or contested their detention with the authorities. Only Cayman Islands had mental health NGOs campaigning for changes to health care on behalf of patients (such as services for children).

**Table 3:** Mental healthcare services

Indicator	Anguilla	Bermuda	BVI	Cayman Islands	Montserrat	TCI
*Services*						
Psychiatric inpatient acute unit	Yes (10 beds)	Yes (23 beds)	Yes (10 beds)	Yes (8 beds)	No	No
Outpatient clinics	Yes	Yes	Yes	Yes	Yes	Yes
Community Mental Health Team	No	Yes	Yes	Yes	Yes	Yes
Child and adolescent mental health services	No	Yes	No	Yes	No	No
Dedicated MH crisis service	No	Yes	No	No	No	No
Talking therapies	Yes (public and private)	Yes (public and private)	Yes (public and private)	Yes (public and private)	Yes (public)	Yes (public and private)
Rehabilitation services	No	Occupational therapy day programme	Occupational therapy day programme	Occupational therapy day programme Long stay facility under construction in 2021	No	No
*Human Resources*						
Psychiatrists	Visiting (1 week/month)	5 consultants and 5 psychiatric residents	2 psychiatrists	4 consultant psychiatrists	Visiting (1 week/2 months)	2 psychiatrists
Psychologists	1 clinical psychologist (community)	6 clinical psychologists	1 clinical psychologist	3 clinical psychologists	1 psychologist	2 clinical psychologists
Psychiatric nurses (inpatient)	12	23	7	10	none	none
Psychiatric nurses (community)	none	12	3	3	2	3
Mental Health Officers	None	6	2	None	1	None
Mental Health Social Workers	None	6	1	None (recruiting)	None	1
Psychiatric aides/Support workers in community	None	6	4	5 inpatient psychiatric aides	None	None
Primary Care clinicians trained in mental health (eg mhGAP)	1 GP (out of six) and 1 nurse	0	4 GPs, 3 nurses	18 GPs (8 public, 10 private)	2 GPs	9 GPs
Treatment coverage	Unknown	Unknown	Unknown	Unknown	Unknown	Unknown
Intersectoral involvement in mental health	Police respond to mental health crises in the community					

Provision of specialist community psychiatric services was available in most sites; in all SIDS except Anguilla community staff, e.g. community psychiatric nurses (CPN), visited service users at home. BVI have a long-standing community mental health team that include unqualified psychiatric aides and dedicated transport supporting CPNs to conduct home visiting. All SIDS reported having outpatient clinics and depot injection clinics. Talking therapies, including Government-funded clinical psychologists, are available in all SIDS. In some SIDS this was well developed, for instance Cayman has the Government-funded Department of Counselling Services, several clinical psychologists as well as many private providers. None of the SIDS had clinical pathways to describe a service user’s optimum journey between primary and secondary healthcare providers; or had clinical guidelines describing how joint working would be undertaken by different services; or how clinical thresholds would be used.

All SIDS reported variable levels of competence amongst their primary care clinicians in the diagnosis and management of mental disorders. They had all implemented WHO’s mental health Gap Action Programme (mhGAP) training [34] for some of their primary care clinicians (often with financial and technical support of PAHO). However, none had a rolling programme of training and support to maintain these skills in a sustainable way. In several SIDS service users and NGOs shared concerns about the lack of awareness and skills in primary care, and this deficit was felt to be a barrier to people seeking treatment.

The Cayman Islands and Bermuda were the only SIDS with any dedicated mental health service for children and adolescents. These included inpatient and outpatient services and clinical psychology. However, all of them (including Cayman and Bermuda) expressed a desire to increase provision for this age group as they saw rising demand and referrals from schools and primary care over recent years. Support is available in schools in all these SIDS through school counsellors, educational psychologists, and in the case of Anguilla, a substance use counsellor. However, in Bermuda accessibility and quality of support differed between private and public schools and in Montserrat several stakeholders, including young people themselves, felt support needed strengthening.

Bermuda has a crisis intervention service available 24/7, including a crisis telephone line that any member of the community can access. The service undertakes assessments and responds to crises with support from the on-call psychiatrist and mental welfare officer. None of the other SIDS had a dedicated service developed for emergency psychiatric presentations in the community. Instead, they typically described a process that involved the police picking up the individual in the community and taking them to the Emergency Room at the general hospital or custody suite at the police station for assessment. An on-call psychiatrist would then be involved in the assessment and decision on admission. In many of the SIDS service users and NGOs reported that admissions could have been prevented if more support in the community existed and alternatives to hospital were available.

Some of the SIDS (Bermuda, BVI, Cayman Islands) offered structured day programmes of occupational activities for either inpatients or those in the community or both. These were delivered from within the hospital estate and were usually led by an occupational therapist and assistants. A 54-bed long term residential mental health facility is under construction in the Cayman Islands. At the time the situational analyses were conducted, none of the mental health services within any of the SIDS appeared to have a strong recovery ethos, in which the wishes and expressed needs of people affected by mental disorders are at the heart of the mental health service [19]. There were limited examples of goal setting and the identification of life aspirations in the work of the mental health services as they supported people with severe mental illness. Service users and NGOs in several SIDS highlighted this as a key weakness of the services.

### Mental health promotion and prevention

In all SIDS, mental health literacy levels were reported to be generally low (Table 4). It was reported that amongst the general population mental illness is largely considered to only encompass severe mental illness such as psychosis, with little awareness around common mental disorders. Likewise, in some SIDS there was low awareness of mental health services, with inpatient care sometimes considered the only source of treatment. Stigma towards people with mental illness was reportedly prevalent in all SIDS. In Bermuda, stakeholders highlighted that attendance at the inpatient facility was often highly stigmatised in the community. Some service users were reportedly hesitant to access outpatient services located on the same site, concerned they would be assumed to be experiencing severe mental illness and would consequently be discriminated against by community members. Respondents in all SIDS highlighted the challenges in ensuring confidentiality and privacy when accessing services in a small population, due to the increased possibility of service users knowing mental health staff outside of their professional context.

**Table 4:** Mental health promotion and prevention

Indicator	Anguilla	Bermuda	BVI	Cayman Islands	Montserrat	TCI
Mental health literacy	Low	Low	Low	Low in some parts of population	Low	Low
Attitudes towards mental illness	High levels of stigma	High levels of stigma	High levels of stigma	Some stigma, reducing	High levels of stigma	High levels of stigma
Suicide prevention strategy	No	No	No	No	No	Yes, 2018
Promotion & prevention in overall mental health strategy	Yes—objectives on suicide prevention, mental health promotion & community participation	N/a	Yes—objectives on mental illness prevention & stigma reduction	Yes—objectives on mental health promotion & prevention	Yes—objectives on mental illness prevention, stigma reduction & intersectoral mental health promotion	Yes—objectives on mental health promotion and prevention
Mental health promotion in education	9 school counsellors, 1 educational psychologist, 1 substance misuse counsellor, 1 school health nurse UNICEF Return to Happiness (150 teachers trained)	Counselling services available Whole School, Whole Community, Whole Child programme promotes mental health and wellbeing	School counsellors UNICEF Return to Happiness has ongoing delivery	17 School counsellors Alex Panton Foundation delivers emotional literacy programme for children (Zippy programme)	School counsellors in secondary schools only UNICEF Return to Happiness	School counsellors UNICEF Return to Happiness
Other mental health promotion activities	None identified	World Mental Health Day activities (week long awareness raising) Annual art exhibition by service users	World Mental Health Day and Mental Health Month activities (awareness-raising talks in schools, information stand in hospital) Programme to increase individual and community resilience following disasters	World Mental Health Day activities (radio, social media, public information, theatre production in 2019) Mental Health First Aid training for over 180 persons in health service, government and private agencies	Weekly radio programme “Enhancing your Mental Health”	Ad hoc activities e.g. public service announcements, radio shows, Facebook posts
Mental health organisations	Red Cross	Bermuda Mental Health Foundation Family support group Salvation Army (drug addiction residential facility Harbour Light and emergency housing shelter) Red Cross	Alzheimer’s Association Red cross	Loud Silent Voices and Alex Panton Foundation (service user organisations) Red Cross	Red Cross	Red Cross

Mental health promotion and prevention activities received some attention at a strategic level. Only TCI had a stand-alone suicide prevention strategy, and suicide prevention was addressed in the broader mental health strategy only in Anguilla. However, other aspects of promotion and prevention featured in the core objectives of mental health strategies for all SIDS. Priority areas included changing attitudes and reducing stigma, promoting emotional wellbeing (particularly among young people) and increasing the detection of mental illness.

All these SIDS carry out some mental health promotion activities, which generally aim to raise awareness and reduce stigma around mental illness. All SIDS focus many activities around World Mental Health Day, including awareness-raising talks in schools, information stands in hospital, social media campaigns, and art exhibitions by service users. In 2019 a play written and performed by Cayman Island residents on the theme of mental health was seen by hundreds of people. Montserrat has a weekly radio programme, “Enhancing your mental health”. In the Cayman Islands, Mental Health first aid training has been provided for over 180 persons in the health service, government and private agencies. In Montserrat, the primary healthcare team proactively undertakes health screening, which includes mental wellbeing screening tools, in the workplace for the employees of larger organisations. Follow-up in the form of group support for those who screen positive is provided by the psychologist.

Since Hurricane Irma, Anguilla, BVI, Montserrat and TCI have participated in UNICEF’s Return to Happiness programme [35]. This intervention addresses the emotional impact of the hurricane amongst children and young people through play, music and drama. In addition, BVI has a broader ongoing mental health promotion programme to build individual and community resilience following the hurricanes. In the Cayman Islands a mental health nurse is integrated into the National Emergency Operations Centre under the Human Concerns Cluster, providing disaster input on mental health. In several SIDS, training on mental health, including legislation, is routinely provided for police and prison officers. Aside from this, limited collaboration with other sectors in mental health promotion or service provision was identified. Apart from Montserrat none of the SIDS had community residential facilities for those with mental disorders.

The role of mental health-related NGOs or civil society organisations is variable; none were identified in Anguilla, Montserrat or TCI. In the Cayman Islands Loud Silent Voices is a support group for service users and their family members and the Alex Panton Foundation (https://alexpantonfoundation.ky/) conducts a range of support and advocacy activities focused on improving the mental health of children and young adults, including in schools. In Bermuda the Salvation Army runs a drug addiction mental health facility, which has a caseload of around 20 individuals and is well linked to statutory health services. The Bermuda Mental Health Foundation conducts awareness-raising activities, and a mental health family support group exists in the BVI. There are Red Cross branches in all UKOTs who provide training on psychosocial support skills for first responders and evacuation shelter staff in the context of emergencies (such as hurricanes).

### Information systems, evidence and research

Health information systems vary across the six SIDS but overall are underdeveloped (Table 5). In general SIDS with lower GDP per capita rely on paper-based systems and those with higher GDP per capita have electronic based systems. Only BVI has a single health information system across public sector primary and secondary providers (including mental healthcare services). No MoHs have any health information system linked to other sectors (e.g. education, social care, welfare benefits), nor use system-wide indicators for monitoring the whole mental health system.

**Table 5:** Information systems, evidence and research

Indicator	Anguilla	Bermuda	BVI	Cayman Islands	Montserrat	TCI
General Health Information systems	Paper-based system at the hospital manually inputted into database	Paper-based system in hospital soon to become electronic	Single electronic system across health system (CELLMA)	Electronic system in government hospital. Government health insurance scheme also holds information	Paper-based system across health system. Plans for electronic system exist	Electronic system in hospital. Not in primary care yet
Mental Health indicators for strategic monitoring	No	No	Some mental health data included in national health monitoring	Paper-based monitoring form used quarterly for limited number of indicators from public and private healthcare providers	No	No
Performance report of the mental healthcare services	No	No	Some mental health data included from primary care, although not as registers (which exist for diabetes, asthma etc.)	Reporting includes some mental health data	Mental health services produce annual report on activity	No
Local mental health research conducted	No	No	No	No	No	No

Aside from the School and Adult Health Surveys, none of the SIDS had conducted local mental health-related research in their populations. This included community surveys to estimate prevalence of mental disorders. The lack of this data limits the ability of the MoHs to plan services and effectively allocate funding to address the disease burden.

## Discussion

### Cross-cutting strengths and weaknesses

This comparative situational analysis has identified several common strengths and weaknesses in the mental health systems of six Caribbean SIDS: Anguilla, Bermuda, BVI, Cayman Islands, Montserrat and TCI. Strengths include national strategic documents existing in most SIDS which are in line with the WHO Mental Health Action Plan. This is in part because of the increasing recognition of the importance of mental health by Governments. There are multi-disciplinary treatment services available in all these SIDS, although those with smaller populations and economies are generally less well resourced. Inpatient units are available in all but one SIDS. There is also community psychiatric service provision which includes both outpatient clinics and some home visiting by CPNs. There are talking therapies available in all SIDS. Low level mental health promotion occurs in all states, particularly awareness-raising through radio shows, school assemblies and social media campaigns, often occurring around World Mental Health Day.

Many common weaknesses in the mental health systems in these SIDS relate to the limitations to mental health personnel and facilities inherent in small populations. There was generally an over-reliance on psychiatric hospital-based services. Most SIDS had minimal specialist input, with two SIDS relying on visiting psychiatrists and all but one lacking a dedicated crisis intervention service. Provision of assessment and treatment services for children and young people were particularly limited and there were some indications of high levels of suicidal thoughts and behaviours in this age group. Integration, joint working and agreed care pathways were often lacking between primary and secondary care. Clinical competencies in primary care staff to diagnose and manage mental disorders were variable and often weak. Recovery-oriented psychosocial rehabilitation services were generally underdeveloped. We observed limited implementation of national strategies, and an absence of key data relating to prevalence of mental disorders, activity of psychiatric services (including time trends analysing shifting care patterns towards community-based care), and levels of stigma and discrimination. The voice of those with lived experience was often lacking in discussions about national mental health policy and practice and there are few organisations established to assist with this. Mental health stigma appears to be pervasive in these SIDS, and may be intensified by the difficulties of anonymity in a small island setting. There were some indications that stigmatizing attitudes may impede access to care. However, there are signs this is changing among younger generations as they have exposure through social media to worldwide changes in attitude. There was some evidence of broader social inequalities in relation to immigration status and race; and signs that stigma and user fees could be barriers to accessing mental healthcare in some SIDS.

### Limitations

Despite these situational analyses being conducted to international best practice standards, there are several limitations. First, we have presented an assessment of each mental health system at one point in time. This is likely to become outdated as services evolve and new contextual factors emerge, particularly during and following the COVID-19 pandemic. All included SIDS had COVID-19 cases and deaths [36]. By April 2021 TCI and Bermuda had over two thousand cases. BVI and Cayman had between 100 and 1000 cases whilst Montserrat and Anguilla had less than 100 cases each. The total combined deaths across all six SIDS was less than 50. Nevertheless, the economy has been substantially impacted, particularly due to the curtailed tourism industry which took most of a year to resume to any degree [37]. Health service and policy developments have been delayed in most SIDS due to disruption caused by the pandemic, although Bermuda’s 2021 Mental Health Code of Practice was published and aims to achieve a recovery-oriented approach to care. Second, for logistical reasons the six situational analyses were conducted consecutively over an 18-month period, potentially limiting comparability between SIDS. However, given the generally slow pace of change in policy and services we believe our results still provide a valuable shared learning opportunity. Third, as there was almost no information available on mental health burden or access to care, we were not able to assess treatment coverage. It was therefore hard to discern whether mental health systems are meeting the needs of the population, including whether access is equitable.

Fourth, the team from the UK may have lacked contextual understanding in their assessment and judgements as they worked cross-culturally. This was mitigated to some extent by close working with the MoHs to sense check observations and findings and compare against international standards where possible. For three SIDS we relied on the expertise of external advisors from the Caribbean region. Finally, engagement with current or previous service users was less than planned due to the lack of local NGOs to facilitate this.

### Findings in context

The challenges of a small resource base, limited direct access to specialist psychiatric services and confidentiality concerns are echoed in small island settings globally, regardless of cultural or economic context [38–40]. However additional needs in the Caribbean region include vulnerability to natural disasters, such as hurricanes. The improvements over recent decades in the mental health systems of these SIDS mirror developments in the whole Americas region, which includes South, Central and North America and the Caribbean [41]. Many Latin American and Caribbean states have developed their mental health systems over the last 30 years, bolstered by increased research capacity and international collaboration [42]. Jamaica and Cuba were early adopters of new models of psychiatric care in the Caribbean region, away from large institutions and towards community-based care [43]. Amongst SIDS included in our analysis BVI has made most progress in this regard, whilst the continuing reliance of the other SIDS on inpatient care is similar to Caribbean islands with lower-income economies, such as St Lucia [39]. Similar to the SIDS in our study, most countries (76%) in the Americas now have stand-alone national mental health policies or plans [41]. However, no SIDS within our study had indicators that monitored implementation of the policy, unlike 75% of countries in the broader Americas region. The proportion of Government spending on mental health, were at similar levels in our SIDS to the Americas. However, despite recent increases in financial input, this is still considered by PAHO to be inadequate to address the disease burden. Gaps in the provision of psychiatric services for children, long inpatient stays for a substantial minority of service users and the lack of social support and community residential facilities were similar in our SIDS to the majority of countries in the region [39, 41]. Mental health promotion programmes are less common in our SIDS than the region, with other countries doing more on suicide prevention, promotion of parental mental health and early child development [41].

### Implications

Mental health system strengthening requires leadership from Governments, Ministries of Health and health authorities. PAHO and UK public health experts should also support SIDS in sharing successful approaches to strengthening mental health systems, for example in relation to mental health personnel and mental health promotion efforts. A fundamental building block for all SIDS is the development of national mental health policies and action plans that include clear indicators of implementation and a governance structure to ensure action is taken. Understanding disease burden is also essential to develop relevant and responsive policies and services. Community surveys to ascertain the prevalence of mental disorders in young people and adults, and access to care, including identifying barriers and inequalities, are needed. To support the timely implementation of mental health action plans, we recommend greater parity on spending with regards to mental health and physical health and the monitoring of total expenditure on mental health in Health and other Government Ministries.

The transition to community-oriented approach is strongly endorsed in the WHO Mental Health Action Plan and is essential to achieve equitable, accessible and effective care provision. We therefore recommend that expenditure and service planning of community mental healthcare be prioritised over tertiary care. To support these endeavours, task-sharing and integration approaches to increase access to primary mental health care should be pursued. Task sharing is widely acknowledged as a fundamental approach to addressing skill shortages in rural and remote settings in high-income countries [44], as well as LMICs. Task-sharing may include the development of clinical competencies among all primary care clinicians for the identification and management of mental disorders (e.g. roll out of the WHO mhGAP training). Options for training new cadres of mental health workers should also be explored, such as mental health officers and psychiatric aides, which are a successful component of mental health systems in Bermuda and BVI. Clear supervision structures, and referral and treatment pathways integrating community, primary and secondary care, are needed to support high quality care provision in all settings. A formal assessment of mental health legislation in all these SIDS against good practice, particularly the UN Convention on the Rights of Persons with Disabilities (CRPD) would identify where improvements in protecting human rights are needed.

As part of a balanced model of care [45], opportunities for increasing access to specialist care should also be explored. This is particularly relevant to SIDS with no resident psychiatrist (Anguilla and Montserrat) and in all SIDS to access sub-specialties such as forensic or child psychiatry. There is some evidence that remote psychiatric assessment and treatment is non-inferior to in vivo psychiatry [46] and telemedicine has been used successfully for other specialities in the Caribbean to access specialists not available on SIDS [47]. Even among small populations, online training and supervision modalities may be an efficient approach to achieving widespread and sustainable coverage [48], taking into account the demanding work schedules of professionals in resource-poor settings and the restrictions related to the COVID-19 pandemic.

We propose that SIDS actively seek out and include the input of those with lived experience in the development, review and implementation of mental health policy and service delivery [49]. This will support efforts to ensure the rights of those with mental disorders are protected, particularly in hospitals or similar institutions. Furthermore, we recommend the development of a culture within services and clinical processes, including psychosocial support, that foster a recovery-oriented approach that assist service users towards living a fulfilling life (as defined by them) [50].

The development of systematic and ongoing programmes of mental health promotion and prevention may have a powerful role in reducing disease burden and promoting access to care [19]. Efforts may include suicide prevention strategies, which should be inclusive of young people. We recommend that Ministries of Education, in collaboration with Ministries of Health and health service authorities, should support all schools to be mental health promoting environments that take a whole school approach [51]. Stigma and discrimination of people with mental disorders in the community can be tackled with evidence-based interventions, such as the UK’s ‘Time to Change’ social marketing campaign [52]. To strengthen resilience against the impacts of future hurricanes, mental health should be embedded within disaster preparedness and planning; programmes implemented in TCI and BVI may be applicable to other settings. Finally, information systems need to be developed to collect and analyse service activity data to monitor and evaluate their impact and effectiveness.

## Conclusion

These situational analyses conducted in six Caribbean SIDS have highlighted some common areas of strength and weakness across their mental health systems. These are similar, in many respects, to mental health systems in larger jurisdictions in the region. Strengths clearly exist in the health systems that address the burden of mental health in these SIDS, but much work is still to be done. The findings and recommendations of these situational analyses are useful for mental health leaders in these SIDS, especially MoHs, in their work to ensure that ‘there is no health without mental health’.

## Supplementary Information

**Additional file 1.** UKOTs mental health situational analysis data collection tool.

## Data Availability

Data sharing is not applicable to this article as no datasets were generated or analysed during the current study.

## Abbreviations

BVI: British Virgin Islands
CPN: Community Psychiatric Nurse
mhGAP: Mental Health Gap Action Programme
MoH: Ministry of Health
PAHO: Pan-American Health Organisation
PHE: Public Health England
PRIME: PRogramme for Improving Mental healthcarE
SAMOA: SIDS Accelerated Modalities of Action
SIDS: Small Island Developing States
TCI: Turks and Caicos Islands
UKOT: United Kingdom Overseas Territory

## References

[1] Rehm J, Shield KD (2019). Global Burden of Disease and the Impact of Mental and Addictive Disorders. Curr Psychiatry Rep.

[2] Suicide in the world (2019). Global health estimates.

[3] The intersection of COVID-19 and mental health. The Lancet Infectious Diseases. 2020;20(11):1217. 10.1016/S1473-3099(20)30797-0 PMC7544473 33038942

[4] Abubakar I, Devakumar D, Madise N, Sammonds P, Groce N, Zimmerman C (2016). UCL- Lancet Commission on Migration and Health. Lancet.

[5] SIDS Accelerated Modalities of Action (SAMOA) Pathway. United Nations General Assembly; 2014.

[6] Suzana M, Walls H, Smith R, Hanefeld J (2018). Achieving universal health coverage in small island states: could importing health services provide a solution?. BMJ glob.

[7] Zahir MZ, Miles A, Hand L, Ward EC (2021). Information and Communication Technology in Schools: Its Contribution to Equitable Speech-Language Therapy Services in an Underserved Small Island Developing State. Lang Speech Hear Serv Sch.

[8] Fitzpatrick KM (2020). Post-traumatic stress symptomatology and displacement among Hurricane Harvey survivors. Soc Sci Med.

[9] Shultz JM, Sands DE, Holder-Hamilton N, Hamilton W, Goud S, Nottage KM (2020). Scrambling for safety in the eye of dorian: mental health consequences of exposure to a climate-driven hurricane. Health Aff (Millwood).

[10] Asugeni J, MacLaren D, Massey PD, Speare R (2015). Mental health issues from rising sea level in a remote coastal region of the Solomon Islands: current and future. Australas.

[11] Gibson KE, Barnett J, Haslam N, Kaplan I (2020). The mental health impacts of climate change: Findings from a Pacific Island atoll nation. J Anxiety Disord.

[12] Roberts E, Bourne R, Basden S (2013). The representation of mental illness in Bermudian print media, 1991–2011. Psychiatr Serv.

[13] Leigh-Hunt N, Bagguley D, Bash K, Turner V, Turnbull S, Valtorta N (2017). An overview of systematic reviews on the public health consequences of social isolation and loneliness. Public Health.

[14] Wang J, Mann F, Lloyd-Evans B, Ma R, Johnson S (2018). Associations between loneliness and perceived social support and outcomes of mental health problems: a systematic review. BMC Psychiatry.

[15] Semrau M, Alem A, Ayuso-Mateos JL, Chisholm D, Gureje O, Hanlon C (2019). Strengthening mental health systems in low- and middle-income countries: recommendations from the Emerald programme. BJPsych Open.

[16] Psychosis and schizophrenia in adults: prevention and management. Clinical guideline [CG178]. NICE (National Institute for Health and Care Excellence); 2014.

[17] Bank W. Disease Control Priorities. Third edition ed. Patel V, Chisholm D, Dua T, Laxminarayan R, Medina Mora ME, editors. Washington D. C.: World Bank Group; 2015. 27227198

[18] Mental Health Action Plan 2013–2020. Geneva: World Health Organisation; 2013.

[19] Patel V, Saxena S, Lund C, Thornicroft G, Baingana F, Bolton P (2018). The Lancet Commission on global mental health and sustainable development. Lancet.

[20] NCDs and COVID-19 in the Caribbean: A call to action. The case for a transformative new NCD agenda. . Healthy Caribbean Coalition 2021.

[21] Situation analysis and priority setting: World Health Organisation; 2018 [Available from: https://www.who.int/nationalpolicies/processes/priorities/en/.

[22] Murphy JK, Michalak EE, Colquhoun H, Woo C, Ng CH, Parikh SV (2019). Methodological approaches to situational analysis in global mental health: a scoping review. Glob Ment Health (Camb).

[23] Gandelman N, Serebrisky T, Suárez-Alemán A (2019). Household spending on transport in Latin America and the Caribbean: A dimension of transport affordability in the region. J Transp Geogr.

[24] Hanlon C, Luitel NP, Kathree T, Murhar V, Shrivasta S, Medhin G (2014). Challenges and opportunities for implementing integrated mental health care: a district level situation analysis from five low- and middle-income countries. PLoS ONE.

[25] World Health Organisation Assessment Instrument for Mental Health Systems (AIMS) Version 2.2. Geneva: World Health Organisation; 2005.

[26] Country Poverty Assessment 2012 Volume 1, Main report.: Government of the Turks and Caicos Islands and the Caribbean Development Bank; 2014.

[27] Population and Housing Census Education Brief. Government of Bermuda Department of Statistics; 2016.

[28] Global School-based Student Health Survey: Montserrat 2008 Factsheet. World Health Organisation; 2008.

[29] Global School-based Student Health Survey: Cayman Islands 2007 Factsheet. World Health Organisation; 2007.

[30] Global School-based Student Health Survey: Anguilla 2016 Factsheet. World Health Organisation; 2016.

[31] Global School-based Student Health Survey: British Virgin Islands 2009 Factsheet. World Health Organisation; 2009.

[32] Cayman Islands Student Drug Use Survey (CISDUS): Risk and Protective Factors Year 9–12. National Drug Council, Cayman Islands; 2016.

[33] Health Survey of Adults in Bermuda. Ministry of Health, Government of Bermuda; 2011.

[34] WHO. mhGAP Mental Health Gap Action Programme: Scaling up care for mental, neurological and substance use disorders. Geneva: World Health Organisation; 2008. 26290926

[35] UNICEF Eastern Caribbean's responses in emergencies. https://www.unicef.org/easterncaribbean/unicef-eastern-caribbeans-responses-emergencies

[36] WHO COVID-19 Dashboard. 2021 [cited 08.03.2021]. https://covid19.who.int/

[37] Asrat B, Schneider M, Ambab F, Lund C (2020). Effectiveness of psychological treatments for depressive symptoms among people living with HIV/AIDS in low-and middle-income countries: A systematic review and meta-analysis. J Affect Disord.

[38] Rimicans K, McInerny T (2018). 52 degrees south: mental health services in the Falkland Islands. BJPsych Int.

[39] Francis KA, Molodynski A, Emmanuel G (2018). Mental healthcare in Saint Lucia. BJPsych Int.

[40] Scholtz M, Harmse A (2018). Psychiatry in Shetland. BJPsych Int.

[41] Atlas of Mental Health of the Americas 2017. Washington, D.C.: Pan American Health Organisation; 2018.

[42] Caldas de Almeida JM (2013). Mental health services development in Latin America and the Caribbean: achievements, barriers and facilitating factors. Int Health.

[43] Almeida JM, Horvitz-Lennon M (2010). Mental health care reforms in Latin America: An overview of mental health care reforms in Latin America and the Caribbean. Psychiatr Serv.

[44] Hoeft TJ, Fortney JC, Patel V, Unützer J (2018). Task-Sharing Approaches to Improve Mental Health Care in Rural and Other Low-Resource Settings: A Systematic Review. J Rural Health.

[45] Thornicroft G, Tansella M (2013). The balanced care model: the case for both hospital- and community-based mental healthcare. Br J Psychiatry.

[46] Drago A, Winding TN, Antypa N (2016). Videoconferencing in psychiatry, a meta-analysis of assessment and treatment. Eur Psychiatry.

[47] Adler E, Alexis C, Ali Z, Allen U, Bartels U, Bick C (2015). Bridging the Distance in the Caribbean: Telemedicine as a means to build capacity for care in paediatric cancer and blood disorders. Stud Health Technol Inform.

[48] Setoya Y, Kestel D (2018). WHO Mental Health Gap Action Programme implementation in the Small Island Development States: experience from the Pacific and English-speaking Caribbean countries. Br J Psych Int.

[49] Service user experience in adult mental health: improving the experience of care for people using adult NHS mental health services. Clinical guideline [CG136]. National Institute for Health and Care Excellence; 2011. 31869024

[50] Slade M, Amering M, Farkas M, Hamilton B, O'Hagan M, Panther G (2014). Uses and abuses of recovery: implementing recovery-oriented practices in mental health systems. World Psychiatry.

[51] Promoting children and young people’s emotional health and wellbeing: A whole school and college approach. London: Public Health England; 2021.

[52] González-Sanguino C, Potts LC, Milenova M, Henderson C (2019). Time to Change's social marketing campaign for a new target population: results from 2017 to 2019. BMC Psychiatry.

[53] Defining Research. NHS Health Research Authority; 2013.

[54] UN World Population Prospects 2019. Total population by sex (thousands): Anguilla]. United Nations Department of Economic and Social Affairs. 2019. https://population.un.org/wpp/DataQuery/.

[55] 2016 Population and Housing Census Report. Government of Bermuda Department of Statistics; 2016.

[56] UN World Population Prospects 2019. Total population by sex (thousands): British Virgin Islands. United Nations Department of Economic and Social Affairs. 2019 [cited 22/02/21]. https://population.un.org/wpp/DataQuery/.

[57] The Cayman Islands’ Compendium of Statistics 2019. Cayman Islands Government Economics and statistics office; 2020.

[58] Intercensal Population Count and Labour Force Survey 2018. Montserrat Statistics Department; 2019.

[59] Vital Statistics Report 2019. Turks and Caicos Islands Government Department of Statistics; 2019.

[60] UN Data. Per capita GDP at current prices- US Dollars: Anguilla. United Nations Statistics Division. 2019 [cited 22/02/2021]. https://data.un.org/Data.aspx?q=GDP+per+capita&d=SNAAMA&f=grID%3a101%3bcurrID%3aUSD%3bpcFlag%3a1.

[61] UN Data. Per capita GDP at current prices- US Dollars: Bermuda. United Nations Statistics Division. 2019. https://data.un.org/Data.aspx?q=GDP+per+capita&d=SNAAMA&f=grID%3a101%3bcurrID%3aUSD%3bpcFlag%3a1

[62] UN Data. Per capita GDP at current prices-US Dollars: British Virgin Islands [Internet]. United Nations Statistics Division. 2019.

[63] UN Data. Per capita GDP at current prices- US Dollars: Cayman Islands. United Nations Statistics Division. 2019. https://data.un.org/Data.aspx?q=GDP+per+capita&d=SNAAMA&f=grID%3a101%3bcurrID%3aUSD%3bpcFlag%3a1.

[64] UN Data. Per capita GDP at current prices- US Dollars: Montserrat. United Nations Statistics Division. 2019. https://data.un.org/Data.aspx?q=GDP+per+capita&d=SNAAMA&f=grID%3a101%3bcurrID%3aUSD%3bpcFlag%3a1.

[65] UN Data. Per capita GDP at current prices- US Dollars: Turks and Caicos Islands. 2019. https://data.un.org/Data.aspx?q=GDP+per+capita&d=SNAAMA&f=grID%3a101%3bcurrID%3aUSD%3bpcFlag%3a1.

[66] Health in the Americas Country Report: Anguilla: Pan American Health Organisation (PAHO); 2013. https://www.paho.org/salud-en-las-americas-2017/?page_id=79#:~:text=Life%20expectancy%20is%2081.3%20years,is%20largely%20dependent%20on%20tourism.

[67] Bermuda’s Population Projections 2016–2026 Government of Bermuda Department of Statistics; 2018.

[68] Stewart LA, Clarke M, Rovers M, Riley RD, Simmonds M, Stewart G (2015). Preferred reporting items for a systematic review and meta-analysis of individual participant data: the PRISMA-IPD statement. JAMA.

[69] Health in the Americas Country Report: Cayman Islands: Pan American Health Organisation (PAHO); 2013. https://www.paho.org/salud-en-las-americas-2017/?page_id=103.

[70] Health in the Americas Country Report: Montserrat: Pan American Health Organisation (PAHO); 2013. https://www.paho.org/salud-en-las-americas-2017/?page_id=139.

[71] Anguilla Population and Housing Census 2011 Preliminary Findings 5. “Who are we? – Ethnic Composition and Religious Affiliation”. Government of Anguilla Statistics Department; 2011.

[72] Bermuda Digest of Statistics. Government of Bermuda Department of Statistics 2020.

[73] VIrgin Islands 2010 Population and Housing Census Report. 2010.

[74] Turks and Caicos Islands Census Turke and Caicos Islands; 2012.

[75] Turks and Caicos Islands Disaster Management Plan. 2019.

[76] Labour Force Survey Report November 2019. Government of Bermuda Department of Statistics; 2019.

[77] British Virgin Islands Labour Force Survey Report 2015. 2015.

[78] Health Survey of Adults and Children in Bermuda. Ministry of Health, Government of Bermuda; 2006.

